# The change in serum Thiol/Disulphide homeostasis after transrectal ultrasound guided prostate biopsy

**DOI:** 10.1590/S1677-5538.IBJU.2016.0114

**Published:** 2017

**Authors:** Hüsnü Tokgöz, Selim Taş, Özlem Giray, Soner Yalçınkaya, Özlem Tokgöz, Cemile Koca, Murat Savaş, Özcan Erel

**Affiliations:** 1 Department of Urology, Antalya Training and Research Hospital, Antalya, Turkey;; 2 Department of Biochemistry , Antalya Training and Research Hospital, Antalya, Turkey;; 3 Department of Radiology, Antalya Training and Research Hospital, Antalya, Turkey;; 4 Department of Biochemistry, Yildirim Beyazit University, School of Medicine, Ankara, Turkey

**Keywords:** Biopsy, Prostate, Oxidative Stress, Ultrasonography

## Abstract

**Objectives:**

The aim of this prospective clinical study was to investigate variations in a novel oxidative stress marker (thiol/disulphide homeostasis) in men who underwent transrectal ultrasound guided prostate biopsy (TRUSB).

**Materials and Methods:**

A total of 22 men undergoing TRUSB of the prostate were enrolled in the study. Patients with abnormal digital rectal examination and/or total prostate specific antigen (PSA) over 4ng/mL underwent TRUSB with 12 cores. Serum samples were obtained before and just after the procedure to evaluate the possible changes in thiol/disulphide homeostasis. Mean age, total PSA and free PSA, prostate volume and histopathological data were also recorded.

**Results:**

Mean age of the study population was 65.05±8.89 years. Significant decreases in native and total thiol levels were documented after the biopsy procedure. However, serum disulphide levels and disulphide/native thiol, disulphide/total thiol and native/total thiol ratios did not significantly change after TRUSB. No correlation was observed between oxidative parameters and total PSA and free PSA levels, prostate volume and histopathology of the prostate. However, mean patient age was significantly correlated with mean native and total thiol levels.

**Conclusion:**

Significant decreases in serum native and total thiol levels related to the prostate biopsy procedure suggest that TRUSB causes acute oxidative stress in the human body. Since our trial is the first in the current literature to investigate these oxidative stress markers in urology practice, additional studies are warranted.

## INTRODUCTION

Prostate cancer is a major health problem globally and the incidence is rising. After the entry into health care the use of conventional ultrasounds, Watanabe et al. described the first transrectal ultrasonography in 1967 (1). During the ‘80s, transrectal ultrasound guided prostate biopsy (TRUSB) became a primary technique for the detection of prostate cancer. Because cancers cannot be accurately visualized by conventional ultrasound, sextant biopsy was developed by Hodge et al. (2). Subsequent investigators proposed obtaining more cores to improve the diagnostic accuracy of TRUSB (3, 4). Over the past decade, a significant number of modifications have been made to the techniques for prostate cancer biopsy. Currently, routine 12-core (extended) biopsy is recommended as an office-based, diagnostic standard for evaluating patients with increasing PSA levels (5). Like all biopsy procedures, TRUSB is an invasive technique and, although rare, may be associated with complications such as acute prostatitis, hematuria and rectal bleeding. As such, it exerts stress on the human body.

Oxidative stress in the body occurs due to the imbalance between antioxidants and reactive oxygen radicals, and leads to various systemic disorders. In 2014, Drs. Erel and Neselioglu developed a novel and automated assay which determined plasma thiol/disulphide homeostasis (6). Thiols may undergo an oxidative reaction via oxidants and form disulphide bonds. A disulphide bond is a covalent bond and the linkage is also called a disulphide bridge. The formed disulphide bonds might be reduced to thiol groups; hence, dynamic thiol/disulphide homeostasis is maintained. So, thiol/disulphide homeostasis may already be regarded as an oxidative stress marker like lipid hydroperoxide, total antioxidant/oxidant status and paraoxonase. It has been shown that an abnormal thiol disulphide homeostasis state is involved in the pathogenesis of various diseases, namely diabetes mellitus, cardiovascular diseases, cancer, rheumatoid arthritis, chronic renal disease, acquired immunodeficiency syndrome (AIDS), liver disease and some neurological disorders (Parkinson disease, Alzheimer disease, Friedreich ataxia, multiple sclerosis and amyotrophic lateral sclerosis) (7-16).

Currently, it remains unclear whether TRUSB causes oxidative stress in the human body. In the current literature, no study has been published that evaluates serum or urinary oxidative stress levels in men who underwent TRUSB. In our study, we investigated the changes in dynamic thiol/disulphide homeostatic state in men undergoing TRUSB for abnormal digital examination or serum total prostate specific antigen (PSA) elevation. In addition, correlation between clinical parameters and levels of oxidative stress markers were also evaluated. Our hypothesis is that TRUSB causes acute oxidative stress in the human body resulting in significant changes in serum levels of oxidative stress markers.

## MATERIALS AND METHODS

The approval of the hospital ethics committee was obtained and 22 males were included in this prospective clinical study. Each patient provided informed consent prior to participation in the study. These patients with abnormal digital rectal examinations or serum total PSA levels of greater than 4ng/mL were previously referred to our clinic for TRUSB. All of the patients were asymptomatic before the procedure. Those with bleeding diathesis or on anticoagulation therapy, with a history of radical prostatectomy or radiotherapy, or with regular drug use were excluded. In addition, men with known systemic diseases like coronary artery disease, diabetes mellitus, liver disease, chronic renal failure, rheumatoid arthritis, or with cancer diagnosis that would affect serum oxidative stress parameters were also excluded. Patients with known neurological disorders were not included in the study. All patients received standard antibiotic prophylaxis of ciprofloxacin 500mg twice daily for 3 days, begun 1 day before the procedure. All prostate biopsies were carried out under TRUS guidance, and an automatic biopsy gun with an 18-gauge needle was used to obtain 12 core biopsies. All of the patients received a rectal enema before the procedure. Patients were examined in the left lateral decubitus position with a 9±5MHz curved-array transrectal probe, and the prostate volume and ultrasonographic appearance in the longitudinal and transverse planes were recorded. Age, histopathologic results of biopsy specimens, serum total PSA and free PSA levels, rectal examination findings of patients and prostate volumes were also recorded.

Just before the biopsy procedure, 10cc of blood was taken from the left arm using a biochemistry tube. One hour after the biopsy, another 10cc blood was taken. Collected serum samples were centrifuged at 1500g for 10 minutes to obtain the supernatant serum fraction and those samples were kept at -80°C until analysis. Thiol/disulphide homeostasis tests were performed in the same way as Drs. Erel and Neselioglu previously described in their paper (6). Two parallel vessels were used for samples. To determine total thiol, 10µL sample was treated with 10µL sodium borohydride in 50% methanol–water solution (v/v; R1), which reduces dynamic disulfide bonds to free thiol groups. Excess reductants were eliminated using 110µL 6.715mM formaldehyde and 10.0mM ethylenediaminetetraacetic acid (EDTA) in Tris buffer 100mM (pH 8.2). For native thiol, 10µL sample was treated with 10µL, 10mM sodium chloride in 50% methanol–water solution (v/v; R1’) and 110µL 6.715mM formaldehyde and 10.0mM EDTA in Tris buffer 100mM (pH 8.2). Then, the DTNB (5,50-dithiobis-[2-nitrobenzoic acid]) solution was added. The first absorbance was taken only after adding R1 and R1’ for total and native thiol, respectively, and the second absorbance was taken after the application of formaldehyde and DTNB solutions, and when the reaction trace forms a plateau (assay duration was approximately 10 min). First absorbance was subtracted from the second. The main wavelength is 415nm, and the secondary wavelength is 700nm. All the chemicals were purchased from Merck Chemicals (Darmstadt, Germany) and Sigma-Aldrich Chemie (Milwaukee, Wisconsin, USA). The amount of dynamic disulphide was calculated by taking half of the difference between total thiol and native thiol groups. In this way, native and total thiols were calculated. Afterwards, serum disulphide levels and the disulphide/native thiol, disulphide/total thiol and native/total thiol ratios were determined.

All analyses were performed using IBM SPSS Statistics Version 20.0 statistical software package (IBM Corp. Released 2011. IBM SPSS Statistics for Windows, Version 20.0. Armonk, NY: IBM Corporation). Categorical variables were expressed as numbers and percentages, whereas continuous variables were summarized as mean and standard deviation and as median and minimum-maximum where appropriate. The normality of distribution for continuous variables was confirmed with the Kolmogorov-Smirnov test. For comparison of two related (paired) continuous variables, the paired samples t-test was used. To evaluate the correlations between measurements, Pearson correlation coefficient was used. The statistical level of significance for all tests was considered to be 0.05.

## RESULTS

**Figure 1:**
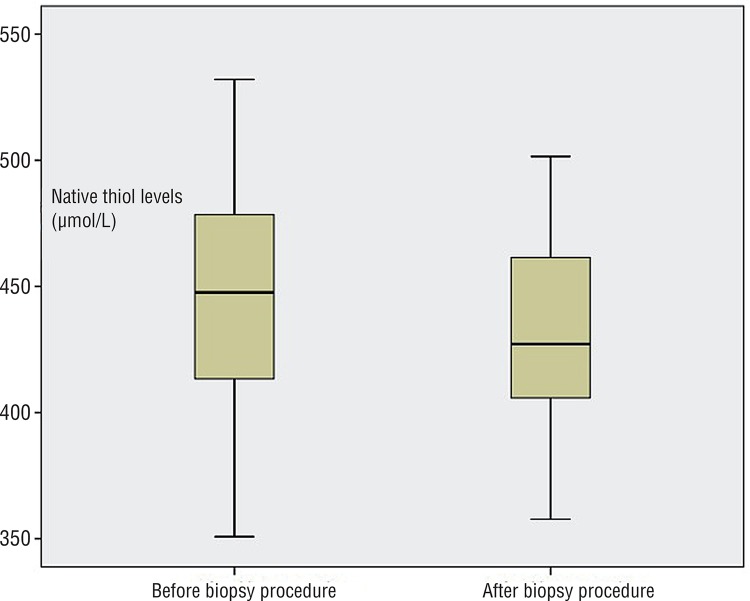
Native thiol levels were significantly lower in men who underwent transrectal ultrasound guided prostate biopsy when compared to the levels just before biopsy (p=0.04, paired samples t test).

**Figure 2:**
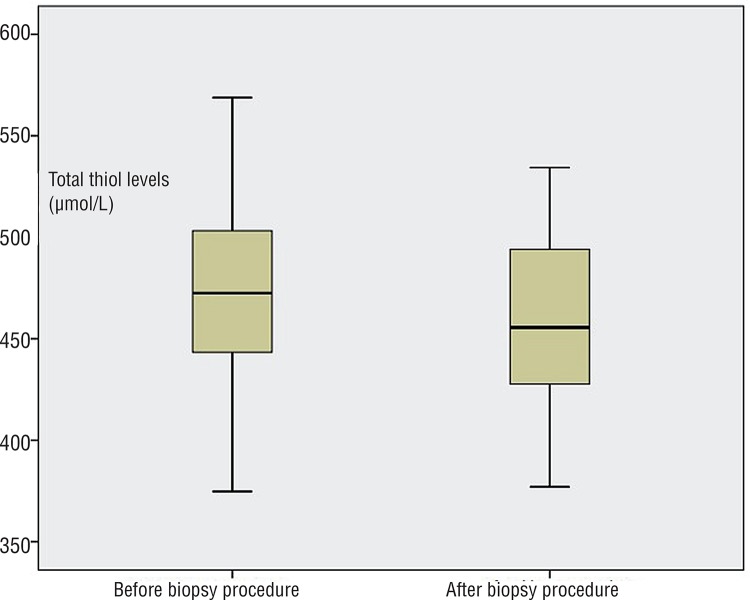
Total thiol levels were significantly lower in men who underwent transrectal ultrasound guided prostate biopsy when compared to the levels just before biopsy (p=0.03, paired samples t test).

Mean age of the study population was 65.05±8.89 years (median age was 66). Mean serum total PSA and free PSA levels, prostate volume and histopathological results of biopsy specimens of all patients are given in Table-1. Significant decreases in mean native and total thiol levels were documented after the biopsy procedure when compared to levels before biopsy (p <0.05, paired samples t test) (Table-2) (Figures 1 and 2). However, mean serum disulphide levels were not significantly changed after the biopsy procedure (p=0.22, paired samples t test) (Table-2). Similarly, the percentage of disulphide/native thiol, disulphide/total thiol and native/total thiol ratios were not significantly changed after TRUSB (Table-2).

**Table 1:** Clinical data of men who underwent transrectal ultrasound guided prostate biopsy.

Variables	
Age*	65.05±8.89
Total PSA (ng/ mL)*	18.25±6.69
Free PSA (ng/ mL)*	3.13±0.77
Prostate volume (cc)*	60.27±29.96
**Histopathology**	
BPH	10 (45.5%)
Chronic prostatitis	2 (9.1%)
ASAP	1 (4.5%)
High grade PIN	2 (9.1%)
Prostate adeno cancer	7
Gleason score 3+3	3 (13.6%)
Gleason score 3+4	1 (4.5%)
Gleason score 4+5	1 (4.5%)
Gleason score 5+4	2 (9.1%)
**Total (number of patients, %)**	**22 (100%)**

**SD =** Standard deviation; **PSA =** Prostate-specific antigen; **Ng/mL =** nanogram per mililiter; **cc =** cubic centimeter; **BPH =** Benign prostatic hyperplasia; **ASAP =** Atypical small acinar proliferation; **PIN =** prostatic intraepithelial neoplasia; *mean±SD

**Table 2:** Changes in the levels of oxidative stress markers after the biopsy procedure with relevant p values.

	Before biopsy	After biopsy	p* value
Native thiol (µmol/L)	441.54±44.88	432.84±36.54	**0.04**
Total thiol (µmol/L)	470.22±47.46	460.17±40.46	**0.03**
Disulphide (µmol/L)	14.32±2.65	13.65±2.68	0.22
Disulphide/ native thiol %	3.25±0.54	3.14±0.47	0.38
Disulphide/ total thiol %	3.04±0.48	2.94±0.42	0.38
Native / total thiol %	93.90±0.96	94.10±0.83	0.39

Data were expressed as mean±standard deviation

Statistically significant p values were written as bold

* Paired samples t test

In the correlation analysis, there was a significant negative correlation between mean patient age and mean pre-biopsy (correlation coefficient=-0.480, p=0.024, Pearson correlation analysis) and post-biopsy native thiol levels (correlation coefficient=-0.593, p=0.004, Pearson correlation analysis). Additionally, a significant negative correlation was also noticed between age and pre-biopsy (correlation coefficient=-0.444, p=0.039) and post-biopsy total thiol levels (correlation coefficient=-0.545, p=0.009). However, no statistically significant correlation was observed between any of the oxidative stress parameters and the total and free PSA levels, prostate volume and histopathology of the prostate (p >0.05, Pearson correlation analysis).

## DISCUSSION

There is no study published which investigates changes in thiol/disulphide homeostasis in urological disorders. However, the investigation of serum levels of different oxidative stress markers is not new. Recently, Ener et al. evaluated the oxidative stress status and antioxidant capacity in patients with painful bladder syndrome, and used serum total antioxidant capacity, total oxidant status, binding capacity of exogenous cobalt to human albumin, serum advanced oxidation protein products, paraoxonase, arylesterase, IgE, and C-reactive protein as oxidative markers (17). Some investigators researched the levels of oxidative markers either in urine or semen (18, 19). When compared to these conventional oxidative stress markers, the novel assay developed by Drs. Erel and Neselioglu provides an easy, relatively cheap, practical, fully automated (optionally manual) spectrophotometric assay for the determination of plasma dynamic thiol/disulphide homeostasis (6). Kundi et al. investigated its use in patients with acute myocardial infarction and found significant changes in serum levels of native thiols, total thiols and disulphide when compared to controls (20). Ates et al. have shown that thiol oxidation increases in prediabetics as well as in type 1 diabetics (21, 22). They also observed a correlation between serum disulphide levels and blood glucose and HbA1c levels. Some other authors also evaluated thiol/disulphide homeostasis in asphalt workers, in patients with masked and primary hypertension, in women with idiopathic recurrent pregnancy loss and pre-eclampsia, and in patients with inflammatory bowel disease (23-30).

This trial is the first to show significant changes in dynamic thiol/disulphide homeostasis in men undergoing TRUSB of the prostate. This means that thiol oxidation increased in men who underwent biopsy of the prostate. It is acknowledged that any biopsy procedure may cause acute oxidative stress in biopsied organs and increase the levels of oxidative stress markers in tissues (31-33). So, trauma to any organ or any systemic disorder may lead to increases in serum levels of oxidative stress parameters. We performed 12-core biopsies to all cases in the study group. It may be considered that serum native and total thiol levels could be correlated with the biopsy core numbers. We hypothesize that increasing the number of biopsy cores may aggravate the thiol oxidation in plasma. To identify whether this is the case or not, another study should be designed to compare serum levels of thiols in men undergoing 12-core and 24-core-saturation biopsies. However, the study groups should be age-matched because age was the only parameter that was significantly correlated with native and total thiol levels in our study. So, older age at biopsy might increase the risk of morbidity and oxidative stress caused by the biopsy procedure. Similarly, as in our study, negative correlations between age and thiol levels were also documented by some other researchers (20, 21). They found inverse correlations between native and total thiol levels and patient age. In their studies, native and total thiol levels decreased with increasing age.

In the study population, out of 22 men, 7 cases were diagnosed with prostate adenocarcinoma. Regarding histopathological diagnosis, when subgroups were formed no significant differences in serum levels of oxidative markers were noticed. But as the cancer group is too small to make reliable statistical analyses, we cannot make a discrete conclusion. Future studies with larger study population are necessary to investigate the correlation between serum thiol levels and histopathology of the prostate.

Potential limitations of this study should be considered. One could reasonably attempt to form an independent control group composed of non-treated healthy subjects. But we thought that it would be more homogenous and reliable to evaluate the same patients in terms of changes in the levels of oxidative stress parameters. Thus, the effect of TRUSB on the human body could be investigated in detail. Another limitation is that our sample size is relatively small, but prospective studies including men with similar ages in larger series may provide more valuable data. We performed two sensitivity tests (repeated measures analysis of variance for both native and total thiol levels). For native thiol levels, we observed a power of 0.520 and for total thiol levels we observed a power of 0.592. Therefore, the sensitivity in this study might be regarded as at least 0.520. In addition, changes in the levels of native and total thiols should also be evaluated in the long term. Thus, consecutive serum sampling at 1-week intervals after the biopsy procedure could be done and alterations or normalization in serum levels of native and total thiols might be determined. However, while the study was ongoing, we did not know which marker levels would vary significantly. So, the focus of our study was assessment of acute oxidative stress. Acute and chronic oxidative stress may lead to different consequences, and evaluation of chronic stress could form the aim of another trial. Finally, we did not compare our data with other oxidative stress parameters such as lipid hydroperoxide, total antioxidant/oxidant status and paraoxonase. However, a MEDLINE search produced many studies related to the use of thiol/disulphide homeostasis as an oxidative stress marker for many clinical disorders (6-16).

In conclusion, an advantage of this novel marker is that it is easily calculated, readily available, and relatively cheap. Significant decreases in serum native and total thiol levels (as the components of thiol/disulphide homeostasis) related to the prostate biopsy procedure suggest that TRUSB causes acute oxidative stress in the human body. Thus, unnecessary biopsies should be avoided. Patient age at biopsy might affect oxidative stress during biopsy. So, men with older age may have an increased risk of oxidative stress caused by the prostate biopsy itself.
